# Maternal Serum Zinc Level and Pre-eclampsia Risk in African Women: a Systematic Review and Meta-analysis

**DOI:** 10.1007/s12011-021-02611-7

**Published:** 2021-02-01

**Authors:** Endalamaw Tesfa, Endalkachew Nibret, Abaineh Munshea

**Affiliations:** 1 Department of Biochemistry, College of Medicine and Health Sciences, Bahir Dar University, Bahir Dar, Ethiopia; 2 Biotechnology Research Institute, Bahir Dar University, Bahir Dar, Ethiopia; 3 Department of Biology, College of Science, Bahir Dar University, Bahir Dar, Ethiopia

**Keywords:** Zinc, Pre-eclampsia, Meta-analysis, Africa

## Abstract

Some studies have reported the association between maternal serum zinc (Zn) levels and pre-eclampsia. However, many studies have reported controversial results. Hence, this systematic review and meta-analysis was planned to generate summarized evidence on the association between maternal serum Zn levels and pre-eclampsia in African women. Four electronic databases such as PubMed, Hinari, Google Scholar, and African Journals Online were searched for studies published in English. Joanna Briggs Institute Meta-Analysis of Statistics Assessment and Review Instrument, and Newcastle-Ottawa Scale were used for data extraction and quality assessment of the included studies. The meta-regression analysis was performed by the Stata 14 software. The standardized mean difference (SMD) values of lipid profiles were computed to assess their association with pre-eclampsia at 95% CI. A total of 12 observational studies were included. The mean values of serum Zn level were significantly lower in pre-eclamptic women as compared with normotensive pregnant women (Zn = 59.40 ± 22.80 μg/dL and 80.24 ± 16.04 μg/dL), respectively. The pooled SMD of Zn was significantly reduced in pre-eclamptic women as compared with normotensive pregnant women with the SMD of −1.45 (95% CI −2.26, −0.65) at 95% CI. In this review, we found that the maternal Zn serum level was significantly reduced in pre-eclamptic women than normotensive pregnant women. This suggests that Zn could be involved in the etio-pathogenesis of pre-eclampsia. However, the specific functions of Zn in pre-eclampsia pathogenesis should be proved in large-scale clinical trial studies.

**Supplementary Information:**

The online version contains supplementary material available at 10.1007/s12011-021-02611-7.

## Introduction

Pre-eclampsia is a multi-system condition occurring after 20 weeks of gestation. It is clinically characterized by new onset of hypertension and proteinuria. Globally, pre-eclampsia is one of the major cause of maternal and prenatal morbidity and mortality [1]. In Ethiopia, pre-eclampsia is also the commonest direct cause of maternal and prenatal deaths [2]. Despite continuing research, the pathogenesis of this disorder is still unclear and delivery of the placenta remains the only cure. Evidences from animal and human studies so far have shown that abnormal placentation, diffuse endothelial cell dysfunction, and increased systemic inflammation contributed to the pathogenesis of pre-eclampsia [3]. Micronutrient deficiencies are common in pregnant women which lead to pre-eclampsia development [4]. Human exposure to excess toxic metals in the environment and deficiency of bio-elements essential for antioxidant defense mechanisms causes oxidative stress, which leads pre-eclampsia [5].

Zinc (Zn) is the second most abundant trace element next to iron which is essential for all living organisms. It exists as a divalent cation and is not redox active under physiological conditions, which explains why zinc performs different physiological roles in a variety of biological processes [6]. Zn is served as the structural components and cofactors of different classes of enzymes [7]. Zinc is an essential trace element for human nutrition that is an integral part of many enzyme systems like DNA polymerase complex [8]. Zn is involved in different signaling pathways [9]. Zn deficiency has been associated with different diseases like pre-eclampsia, different types of cancer [10], cardiovascular disease [11, 12], skin disease, immunity [9, 13], aging [13], and infection [14]. Zn is important for normal pregnancy and fetal development [15, 16] and its deficiency during pregnancy or in early childhood period leads to stunting, mental retardation, and delayed sexual maturity [8, 17].

Many studies have tried to explore the association between the serum levels of Zn in pre-eclampsia, but many of them have reported conflicting results. Some studies have shown significant low levels of Zn in pre-eclamptic women as compared to normotensive pregnant women [18–20]. However, some studies have found that the serum Zn concentration was not varying significantly between the two groups [21–23]. Therefore, the present systematic review and meta-analysis was planned to generate summarized evidence on the association between maternal serum Zn levels and pre-eclamptic in African women.

## Methods and Materials

### Protocol and Registration

This review protocol is registered at the National Institute for Health Research: PROSPERO international prospective register of systematic reviews with registration number CRD42020203746 at https://www.crd.york.ac.uk/prospero/#recordDetails.

### Study Design and Search Strategy

A systematic review and meta-analysis of published studies were conducted to assess the association between maternal serum levels of zinc and pre-eclampsia. We searched the following databases: PubMed, Hinari, African Journals Online (AJOL), and Google Scholar. The search was done by using Medical Subject Heading (MeSH) terms: “Serum, Zinc, Zn, trace elements, Pre-eclampsia AND Africa” separately or in combination. All published articles up to August 31, 2020, were retrieved and assessed for their eligibility for their inclusion in this review. Preferred Reporting Items for Systematic Reviews and Meta-Analyses (PRISMA) guideline was utilized to conduct this systematic review and meta-analysis.

### Eligibility Criteria

#### Inclusion and Exclusion Criteria

1. Studies conducted in African pre-eclamptic women were included.
2. Studies with case-control, comparative cross-sectional, and cohort designs were included.
3. Articles that report pre-eclampsia as an outcome variable were included.
4. Published and unpublished articles written in English were included.
5. Studies reporting serum levels of Zinc in mean and standard deviation were included.
6. Conference papers, editorials, reviews, and randomized control trials were excluded.

### Study selection and screening

**Fig. 1:**
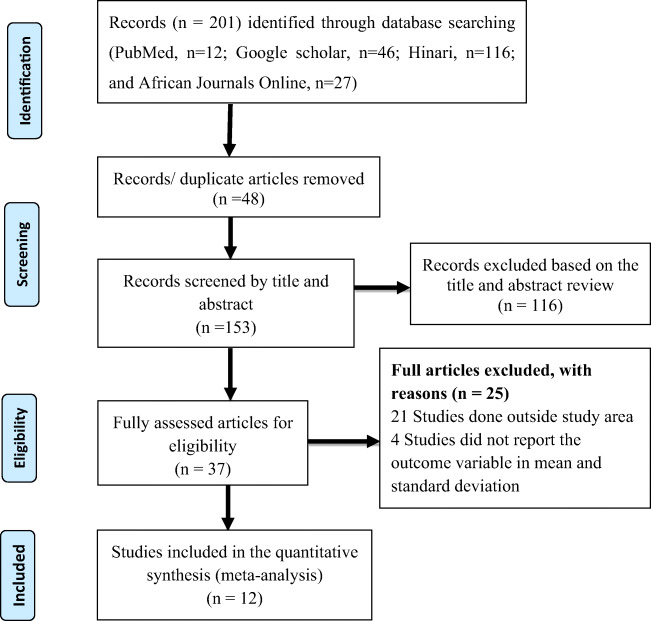
Flow diagram showing the eligibility of studies included in this review

All citations identified by our search strategy were exported to EndNote-X9 and duplicate articles were removed. And then the titles and abstracts of the identified articles were screened by two independent reviewers, and eligible studies were included for further review. The full texts of selected articles were retrieved and read thoroughly to ascertain the suitability prior to data extraction. In case of disagreement between the two reviewers, discussion was held to reach consensus and the third reviewer was consulted. The search process was presented in the PRISMA flow chart that clearly shows the studies that were included and excluded with reasons of exclusion (Fig. 1) [24].

### Definition of Outcome Interest

The primary outcome of this study was to evaluate the association between the maternal serum levels of Zn with pre-eclampsia in African women.

- Hypertension is defined as the systolic blood pressure ≥140 mmHg (SBP) and/or diastolic blood pressure ≥90 mmHg and measured at least two times within 4-h interval.
- Proteinuria: urinary protein excretion of ≥300 mg/24 h urine sample or ≥1*+* on qualitative dipstick examination or a total protein: creatinine ratio ≥30 mg/mmol (or ≥0.3 when both are measured in mg/dL).
- Gestational hypertension: hypertension diagnosed after 20 weeks of gestation.
- Pre-eclampsia is defined as hypertension plus proteinuria after 20 weeks of gestation.
- Eclampsia: seizures in women with hypertension that cannot be attributed to other causes [25].

### Quality Assessment

For case-control studies, we used the Newcastle-Ottawa Scale (NOS) to assess the quality of the included studies while for cross-sectional studies, the modified version of NOS was used to assess the quality of the studies for inclusion [26]. The NOS included 3 categorical criteria with a maximum score of 9 points. The quality of each study was rated using the following algorithm scoring: ≥6 points was considered “Good,” 4 to 5 points was considered “Fair,” and ≤3 point was considered “Poor” quality study. Accordingly, in order to improve the validity of this systematic review result, we only included primary studies with fair to good quality [26].

### Data Extraction Process

The data extraction was done using a tool developed by the 2014 Joanna Briggs Institute Reviewers’ Manual data extraction form [27]. The abstract and full-text were reviewed by the two independent reviewers. Data extraction includes author’s name, publication year, study country, study design, sample size, number of cases and controls, mean age, mean gestational age, mean body mass index (BMI), mean SBP, mean DBP, and mean Zn level. Zn results reported other than μg/dL were changed by multiplying their conversion factors.

### Data Analysis

The data were entered into Microsoft Excel and the meta-analysis was performed using the Stata 14 software and SPSS. Forest plot of SMD was used to assess the strength of association between the serum levels of Zn and pre-eclampsia at 95% CI. The SMD is the ratio of the mean difference to the pooled standard deviation. Standard error of mean (SEM) calculated by the formula SEM= SD/√*n*. Subgroup analysis was done by country (Nigeria, Sudan, Egypt, Kenya, and Zambia). Variables like maternal age, gestational age, BMI, mean SBP, mean DBP, and mean Zn level were analyzed.

### Heterogeneity and Publication Bias

Statistical heterogeneity was estimated through Cochrane’s *Q*, *I*^2^ statistic, and *P* value. *I*^2^ statistic values < 25%, 25–50%, and ≥50% were used to declare the heterogeneity test as low, medium, and high heterogeneity. In this review, random effect model (REM) was used for analysis. To cope with the reasons of heterogeneity, subgroup analysis and sensitivity test were performed. Publication bias was assessed through Egger’s test and funnel plot.

## Results

### Study Selection

A total of 201 articles were retrieved through electronic search by using different search terms of which 153 article were eligible for title and abstract assessment after removal of 48 duplicate records. Out of 153 articles screened for eligibility, 116 records were excluded by their title and abstract assessment. A total of 33 articles underwent full-text assessment for eligibility, and 25 studies were excluded due to different reasons (21 articles were done outside the study area and four studies did not report the result in mean and standard deviation).

### Study Characteristics

In this review, a total of 12 studies were included [5, 19–21, 23, 28–34]. Nine of them were case-control [19–21, 29–34] and three studies [5, 23, 28] were cross-sectional. Studies that have been conducted in Africa and published up to August 31, 2020, were included. Five studies were done in Nigeria, three in Sudan, two in Egypt, and the other two studies were done in Kenya and Zambia. In this review, a total of 1599 pregnant women were included (775 cases and 824 controls) (Table 1).

**Table 1:** Characteristics of research articles included in the systematic review and meta-analysis (*N*= 12)

No	Authors	Country	Study design	Total	PE	NP	Zn PE, μg/dL (mean ± SD)	Zn NP, μg/dL (mean ± SD)	Quality score
1	Mohamed et al. 2019 [20]	Egypt	Case-control	50	25	25	60.8 ± 12.8	95.70 ± 10.40	5 points
2	Pulei et al. 2018 [33]	Kenya	Case-control	108	54	54	64.74 ± 24.2	69.98 ± 22.89	6 points
3	Elmugabil et al. 2016 [21]	Sudan	Case-control	100	50	50	108 ± 23.21	102 ± 27.02	6 points
4	Chababa et al. 2016 [28]	Zambia	Cross-sectional	98	41	57	89.17 ± 47.2	76.20 ± 35.23	6 points
5	Ikaraoha et al. 2016 [19]	Nigeria	Case-control	209	59	150	45.8 ± 9.70	68.2 ± 10.10	5 points
6	Onyegbule et al. 2016 [32]	Nigeria	Case-control	102	54	48	54.08 ± 3.92	79.52 ± 11.97	6 points
**7**	Eldaem et al. 2016 [29]	Sudan	Case-control	200	100	100	17.77 ± 23.2	99.24 ± 27.02	6 points
8	Hassan et al. 2014 [31]	Sudan	Case-control	201	122	79	49.4 ± 17.0	90.3 ± 16.80	6 points
9	Akinloye et al. 2010 [5]	Nigeria	Cross-sectional	89	49	40	56.24 ± 9.16	61.47 ± 5.23	5 points
10	El-Moselhy et al. 2010 [30]	Egypt	Case-control	200	100	100	60.81 ± 9.74	95.7 ± 12.41	6 points
11	Ugwuja et al. 2010 [34]	Nigeria	Case-control	80	40	40	65.20 ± 63.70	71.09 ± 67.23	5 points
12	Enebe et al. 2020 [23]	Nigeria	Cross-sectional	162	81	81	40.80 ± 39.0	53.50 ± 80.0	6 points

*NP* normal pregnant, *PE* pre-eclampsia, *SD* standard deviation, *μg/dL* microgram per deciliter, *Zn* zinc

### Association of Different Variables with Pre-eclampsia

In this analysis, we compared the mean values of variables among pre-eclamptic and normal control groups. Statistical significant difference was not observed between the two groups with regard to the mean values of women’s age, gestational age, BMI, and DBP. However, statistical significant differences were found in the mean values of SBP and serum Zn level between the two groups [5, 19–21, 23, 28–34] (Table 2).

**Table 2:** Paired sample test association of variables with PE

S. No	Variable	Studies	Cases (*N*=775)	Controls (*N*=824)	*P* value
1	Age in year (mean ± SD)	6	27.78 ± 2.72	27.74 ± 1.48	0.974
2	GA in week (mean ± SD)	4	31.70 ± 7.06	31.56 ± 6.92	0.504
3	BMI (mean ± SD)	4	28.61 ± 1.92	27.66 ± 2.20	0.057
4	SBP (mean ± SD)	5	160.27 ± 14.61	115.38 ± 3.89	0.006**
5	DBP (mean ± SD)	5	83.08 ± 40.62	72.95 ± 3.43	0.604
6	Zinc (μg/dL)	12	59.40 ± 22.80	80.24 ± 16.04	0.016**

*BMI* body mass index, *DBP* diastolic blood pressure, *GA* gestational age, *SBP* systolic blood pressure, *SD* standard deviation, *μg/dL* microgram per deciliter

**Statistically significant at *P*<0.05

### Association of Zinc with Pre-eclampsia

**Fig. 2:**
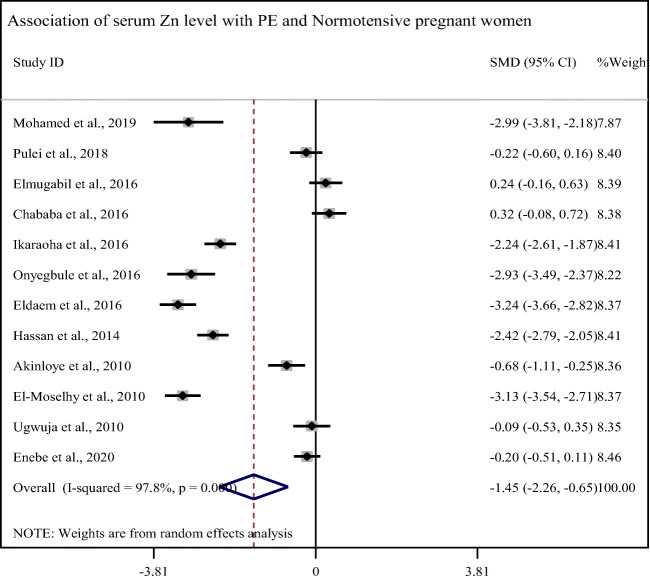
Forest plot of SMD of serum Zinc level in pre-eclampsia

In this sub-categorical analysis, 12 studies were included to compare the serum Zn level between pre-eclampsia and normotensive pregnant women. Seven of the included studies showed significantly lower serum levels of Zn in pre-eclamptic than normotensive pregnant women [5, 19, 20, 29–32]. However, five studies showed non-significant association between the serum levels of Zn and pre-eclampsia [21, 23, 28, 33, 34]. Pooled meta-regression analysis showed that the serum levels of Zn is statistically decreased in pre-eclamptic women as compared with normotensive pregnant women with a pooled SMD of −1.45 (95% CI −2.26, −0.65) (Fig. 2).

### Sensitivity Analysis and Publication Bias

**Fig. 3:**
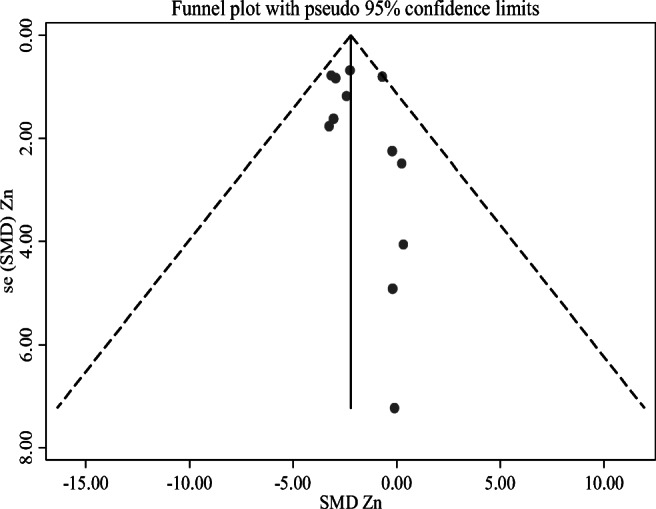
Funnel plot of serum levels of Zinc in pre-eclampsia

A sensitivity test was done by omitting one study at a time to assess the stability of the results. There was no significant change in the pooled SMD after excluding one of the studies at 95% CI (Supporting file 1). This means there is no individual study that excessively influence on the pooled effects of the serum Zn level and risk of pre-eclampsia. Funnel plot did not show evidence of publication bias between maternal serum Zn levels and pre-eclampsia (Fig. 3). And also, Egger’s test did not show evidence of publication bias and its *P* value was 0.319.

## Discussion

This is the first compressive systematic review and meta-analysis of studies evaluating the association between the serum levels of Zn with pre-eclampsia as compared to normotensive pregnant women in Africa. In this study, maternal age, gestational age, and DBP were comparable between the two groups and these variables showed non-statistical significant association with pre-eclampsia. The mean SBP and mean maternal serum level of zinc were significantly associated with pre-eclampsia as compared to normotensive pregnant women.

In this review, the mean serum zinc level was significantly reduced in pre-eclamptic women as compared to normotensive pregnant women (59.40 ± 22.80 μg/dL and 80.24 ± 16.04 μg/dL), respectively, and its pooled SMD of Zn was (SMD= −1.45, 95% CI −2.26, −0.65). Similar finding was reported in systematic review and meta-analysis conducted in China [35]. In the studies conducted in Saudi Arabia and India, the serum levels of zinc were also significantly lower in pre-eclamptic women as compared with normotensive pregnant women [18, 36]. Zn deficiency is observed in almost 17% of the global population and affects many organ systems which causes malfunction of both the humoral and cell-mediated immunity [37].

Zn is an essential metal and served as the structural components of many structural proteins and the cofactors for several metalloenzymes [38]. Zn is required during pregnancy for placental morphogenesis and maternal blood pressure regulation and its deficiency impairs fetal growth and blood pressure abnormalities. Animal model experimental studies proved that zinc deficiency leads to abnormal placental morphogenesis and maternal blood pressure [39]. Zn also involved in the cellular neuronal systems and its deficiency may severely affect the homeostasis of a biological system [40]. Available data suggested that Zn had a significant function in spermatogenesis, embryogenesis, fetal development, and maintaining deoxyribonucleic acid (DNA) integrity. High or low level of zinc concentration might have significant impact on sperm and egg development [41].

Zn is involved in stability of membrane structure, DNA transcription, protein transport systems, and signaling pathways [42]. It is a multipurpose trace element, which binds to more than 300 enzymes and 2000 transcriptional factor proteins [37]. Zn is required for the synthesis of protein and collagen, which contributes to wound healing and a healthy skin [37]. Zn deficiency results major health consequences such as severe defects in growth, development, and proper functioning of the reproductive, immune, and neurosensory systems and in behavior [43]. Its deficiency may contribute for tumor progression via increased expression of the nuclear factor kappa B (NF-κB)-dependent pro-tumorigenic cytokines and impaired in Zn homeostasis which has been observed in different forms of cancers [10]. Inadequate nutritional intake, decreased absorption, or increased loss of zinc all are responsible for zinc deficiency [12].

Increased production of free radicals and reduced levels of some trace elements disrupt the antioxidant defense mechanisms, which contributed oxidative stress. In this review, the serum Zn level was decreased in pre-eclamptic women than normal controls; the mechanism how Zn deficiency causes pre-eclampsia is not clearly understood. However, animal model studies suggested that Zn deficiency may induce high blood pressure by promoting sodium reabsorption by increasing Na^+^-Cl^−^ cotransporter expression [44]. Zn deficiency in prenatal and postnatal rats induces blood pressure derangement accompanied by cardiovascular and renal morphological and functional changes [45].

This systematic review and meta-analysis generates pooled data that showed the association between serum levels of Zn in African pre-eclamptic women. In addition, this review served as baseline information for further study but this study is not free from limitations. The first limitation was related to our literature search strategy, and we included articles published in English, and this could lead to reporting bias. Most of the included studies were from Nigeria, and this may influence its generalizability. Moreover, presence of high statistical heterogeneity among the included studies would decrease the evidence of the review.

## Conclusion

In this systematic review and meta-analysis, the mean serum levels of Zn was significantly reduced in pre-eclamptic women as compared with normotensive pregnant women. Pooled SMD of serum Zn level was also significantly reduced in pre-eclamptic women as compared with normal pregnant women. Thus, Zn could play certain roles in the pathogenesis of pre-eclampsia. However, concrete evidences on the functions of Zn and risk of pre-eclampsia pathogenesis in African women would require large-scale studies.

## Supplementary Information

ESM 1 S1 Sensitivity test (word document) (DOCX 20 kb).

## Data Availability

All data pertaining to this study are contained and presented in this document and in the supplementary files.

## Abbreviations

BMI: Body mass index
CI: Confidence interval
DBP: Diastolic blood pressure
DNA: Deoxyribonucleic acid
GA: Gestational age
JBI-MAStARI: Joanna Briggs Institute Meta-Analysis of Statistics Assessment and Review Instrument
μg/dL: Microgram per deciliter
NOS: Newcastle-Ottawa Scale
PE: Pre-eclampsia
PRISMA: Preferred reporting items for systematic reviews and meta-analyses
RR: Risk ratio
SBP: Systolic blood pressure
SD: Standard deviation
SEM: Standard error of mean
SMD: Standardized mean difference
Zn: Zinc
