# Supplementary material for: Maternal Serum Zinc Level and Pre-eclampsia Risk in African Women: a Systematic Review and Meta-analysis
Source: Biol Trace Elem Res. 2021 Feb 1;199(12):4564–71. doi: 10.1007/s12011-021-02611-7 (PMC8516764; doi:10.1007/s12011-021-02611-7)
Supplement: Supplementary file 1 — S1 Sensitivity test (word document) (DOCX 20 kb). [file 12011_2021_2611_MOESM1_ESM.docx]

**Sensitivity test of serum levels of Zn^2+^ in African pre-eclamptic women**

Fig.1 Sensitivity test for serum Zn^2+^ in pre-eclampsia and normotensive pregnant women
